# Synthesis and Thiol-Ene Photopolymerization of Bio-Based Hybrid Aromatic–Aliphatic Monomers Derived from Limonene, Cysteamine and Hydroxycinnamic Acid Derivatives

**DOI:** 10.3390/polym16233295

**Published:** 2024-11-26

**Authors:** Ricardo Acosta Ortiz, Jorge Luis Robles Olivares, Roberto Yañez Macias

**Affiliations:** Centro de Investigación en Química Aplicada, Department of Macromolecular Chemistry and Nanomaterials, Blvd Enrique Reyna #140, Saltillo 25294, Mexico; jorge.robles.d20@ciqa.edu.mx (J.L.R.O.); roberto.yanez@ciqa.edu.mx (R.Y.M.)

**Keywords:** poly(amide–thioethers), coumaric acid, ferulic acid, phloretic acid, thiol-ene photopolymerization

## Abstract

Three novel bio-based monomers were synthesized through an amidation reaction involving allylated derivatives of coumaric, ferulic and phloretic acid and a diamine obtained from a thiol-ene coupling reaction between limonene and cysteamine. The monomers containing the enone bond of the cinnamic moiety underwent photoisomerization and photocycloaddition reactions upon UV light irradiation. All three monomers were photocured via thiol-ene photopolymerization using a glycerol-derived trifunctional thiol, resulting in fully bio-based poly(amide–thioether)s. The polymers derived from monomers that contain the enone bond exhibited glass transition (T_g_) temperatures of 85 °C when a stoichiometric ratio of the thiol was used, whereas polymers in which an excess of thiol was used exhibited T_g_ temperatures of 61 and 74 °C. The higher T_g_ of the synthesized polymers, compared with other reported polymers produced from thiol-ene photopolymerizations, was attributed to the combination of the aromatic rings of the cinnamic moiety and the cycloaliphatic ring of limonene, as well as the presence of the amide groups in the polymer, which can induce hydrogen bonding. The development of high T_g_ polymers from bio-based monomers through thiol-ene photopolymerization represents a significant advancement in the polymer synthesis sector, offering an improved performance and sustainability.

## 1. Introduction

The growing global environmental awareness, driven by concerns about fossil fuel emissions and the escalating plastic waste crisis, has prompted a quest for sustainable materials that are both eco-friendly and bio-based [1,2]. In the field of polymeric materials, the development of bio-based alternatives with comparable or superior physical and mechanical properties to those of petrochemical-derived materials remains an ongoing objective [3]. Various bio-based building blocks, particularly those derived from biomass, hold promise for addressing environmental challenges while advancing the field of sustainable polymers [4,5,6]. For instance, lignin derivatives such as hydroxycinnamic acid compounds serve as excellent candidates for preparing bio-based monomers due to the aromatic ring in their structure, which imparts excellent thermal and mechanical properties to the resulting polymers. p-Coumaric acid exhibits antioxidant properties attributed to the phenolic group within its structure [7]. This acid is naturally present in plants, cereals, fruits and vegetables [8] and possesses anti-inflammatory [9], anti-cancer [10,11], anti-diabetic [12,13] and anti-melanogenic properties [14,15]. As a starting material, p-coumaric acid is particularly attractive for the synthesis of bio-based monomers, leading to materials with favorable mechanical and thermal properties due to the rigid structure conferred by the aromatic nucleus conjugated with the double bond. Ferulic acid, another lignin derivative found in wheat, rice and sugarcane [16], shares similar antioxidant and anti-inflammatory properties with p-coumaric acid [17]. It also holds potential benefits for skin health as an antiaging agent [18]. Ferulic acid is a precursor for obtaining vanillin through decarboxylation [19]. Phloretic acid is another naturally occurring compound [20,21,22] prepared by reducing the double bond of coumaric acid [23]. This compound also exhibits similar biomedical properties to that of coumaric acid [20].

Several research groups have exploited the advantages of hydroxycinnamic acid derivatives in preparing bio-based polymers. For example, Takada and collaborators [24] reported the preparation of polyesters derived from coumaric, caffeic and ferulic acids. The ability of cinnamic acid derivatives to undergo the E-Z isomerization of the α, β−conjugated double bond (enone), as well as [2+2] a cycloaddition reaction forming dimers, was investigated in this work. Hiraishi et al. [25] reported the preparation of polyesters derived from caffeic and coumaric acid. These polymers were tested as bioadhesives in medical and dental applications. Poly(ether-amide)s were synthesized in three steps using coumaric, ferulic and sinapic acids along with several aliphatic α−ω diamines [26]. The poly(ether-amide)s were designed as bio-based mimics to aramids such as Kevlar^®^ or Nomex^®^.

Thiol-ene photopolymerization is a well stablished technique widely employed in material science [27,28,29,30]. Due to its exceptional reactivity properties, this technique has been considered as a “Click” reaction. Thiol-ene photopolymerization involves the reaction of a compound with two or more thiol groups with another compound bearing two or more double bonds, in the presence of a photoinitiator, following a step-growth mechanism. The reaction proceeds rapidly and quantitatively even at room temperature. Unlike some other polymerization methods, thiol-ene photopolymerizations are not significantly affected by the presence of oxygen and humidity. This robustness allows for reliable processing and curing in various environments. The produced polymers present high uniformity in the crosslink density due to the step-growth mechanism involved. This technique has been utilized to prepare polythioethers from lignin derivatives. For instance, Ge and collaborators [31] prepared a bio-based degradable monomer by reacting allylated vanillin and pentaerythritol to form an allylated acetal. This compound was reacted with commercially available multifunctional thiols such as trimethylolpropane tris (3-trimercaptopropionate) (TMPTMP) and pentaerythritol tetrakis (3-mercaptopropionate) (PTMP) to form polythioethers with T_g_s in the range of 30–50 °C. Xue and collaborators [32] synthesized a family of α,ω di-eugenol derivatives connected by an aliphatic chain of varying length. These compounds were polymerized via thiol-ene photopolymerization using TMPTMP and PTMP as comonomers. The produced polythioethers exhibited T_g_ values ranging from −1 °C to 17 °C, depending on the length of the aliphatic chain and the type of multifunctional thiol. The storage moduli of the polymers were significantly reduced due to the inherent flexibility of polythioethers in addition to the flexibility imparted to the polymers by the connecting aliphatic chain in the monomers. Shibata et al. [33] performed allylation reactions of phenolic compounds such as *p*-coumaric acid and caffeic acid to obtain a diallylated p-coumaric acid and triallylated caffeic acid. These monomers were photopolymerized along with PTMP in a 1:1 and 1:2 ratio considering that both the allyl double bond and the enone double bond can react with the thiol via the thiol-ene photopolymerization mechanism, producing polythioethers with T_g_ values of −2.8 °C and 4.6 °C for the diallylated coumaric acid and triallylated caffeic acid, respectively. Notably, in this investigation, it was found that the [2+2] dimerization reaction did not occur during the thiol-ene photopolymerization. The same group [34] reported the synthesis of a vanillin-derived monomer via an aldolization reaction of vanillin with acetone using an acid catalyst. The produced intermediate was further allylated to obtain the diallyl derivative DADVAT. Guzman and collaborators [35] reported a method to dimerize eugenol via oxidative coupling to obtain bis-eugenol, employing potassium ferricyanide as a catalyst. This compound was further allylated and then photopolymerized with three different thiols, producing polymers with T_g_ values in the range of −6 °C to 26 °C. Aoyagi and collaborators [36] utilized glutamic acid and tyramine as natural raw materials to prepare allylated bio-based monomers. These monomers were photopolymerized along with PTMP, finding that the T_g_ of the photocured films derived from these monomers were −0.11 °C and 2.2 °C for the allylated glutamic acid and the allylated tyramine-derived polymers, respectively, indicating that the aromatic ring in the tyramine monomer induced higher rigidity than the aliphatic glutamic acid monomer.

Continuing with the development of bio-based monomers, this study aimed to investigate the chemical, thermal and viscoelastic properties of polymers synthesized from three hybrid aromatic–aliphatic monomers. The monomers were synthesized via amidation reactions between the allylated derivatives of coumaric acid, ferulic acid and phloretic acid and a diamine obtained through a thiol-ene coupling reaction between limonene and cysteamine. These monomers were photopolymerized in the presence of a bio-based trifunctional thiol derived from glycerol. Following thiol-ene photopolymerization, the resulting poly(amide–thioether)s were analyzed using differential scanning calorimetry (DSC), dynamic mechanical analysis (DMA) and thermogravimetric analysis (TGA).

## 2. Materials and Methods

### 2.1. Reagents

**Scheme 1:**
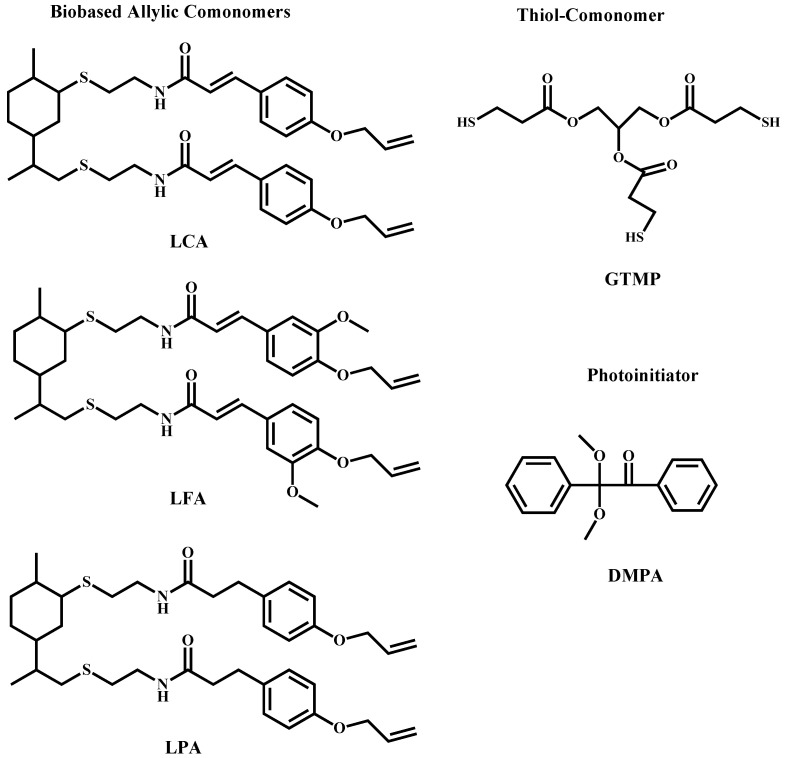
Chemical structures of the synthesized bio-based allylic comonomers, the trifunctional thiol used as comonomer and the photoinitiator.

(*R*)-(+)-4-Isopropenyl-1-methyl-1-cyclohexene (limonene) 97%, 2-aminoethanethiol (cysteamine) hydrochloride 98%, trans-4-hydroxycinnamic acid [coumaric acid, (CA)] 98%, *trans*-4-hydroxy-3-methoxycinnamic acid [ferulic acid, (FA)] 99%, 3-(4-hydroxyphenyl) propionic acid [phloretic acid, (PA)] 98%, boric acid (99.5%), 1,2,3-propanetriol (glycerol) 99.5%, 3-mercaptopropionic acid (99%) (MPA), tetrabutylammonium bromide (TBAB) 99%, *p*-toluenesulfonic acid monohydrate 98%, phenylbis(2,4,6-trimethylbenzoyl)phosphine oxide (BAPO) 97% and 2,2-dimethoxyphenyl acetophenone (DMPA) 99% were all purchased from Sigma-Aldrich (Toluca, Mexico) and used as received. The chemical structures of the synthesized monomers, comonomer and photoinitiator are depicted in Scheme 1.

### 2.2. Synthesis of (E)-3-(4-Allyloxy)phenyl) Acrylic Acid (ACA)

The synthesis of ACA was carried out in three stages. First, to protect the carboxylic acid, CA was esterified with ethyl alcohol using the following method: 20 g (0.1203 mol) of CA and 100 mL of ethanol were charged in a 250 mL three-necked round-bottom flask fitted with condenser, thermometer and magnetic stirrer. Then, 0.078 mL of concentrated hydrochloric acid was added. The mixture was refluxed for 24 h and then cooled to room temperature. The excess ethanol was removed by rotary evaporation and the resulting residue was purified by column chromatography using a gradient mixture of hexane/ethyl acetate as the eluent. The product ethyl coumarate (ECA) was obtained as a white solid with an 88% yield. In the second stage, ECA was alkylated with allyl bromide via the Williamson etherification reaction to produce the allylated derivative (AECA). The synthetic method employed was as follows: 26.35 g (0.137 mol) of ECA and 150 mL of toluene were charged into a 100 mL three-necked round-bottom flask fitted with a condenser, thermometer and magnetic stirrer. Then, 29.07 g (0.274 mol) of sodium carbonate was added to the reaction mixture, followed by the dropwise addition of 33.18 g (0.274 mol) of allyl bromide. Next, 3.09 g (0.009 mol) of TBAB was added as a phase transfer catalyst and the resultant mixture was refluxed for 24 h. The obtained mixture was filtered, and the organic phase was washed with distilled water (3 × 60 mL) and subsequently dried with anhydrous sodium sulfate. After filtration of the solid and evaporation of the solvent, the produced residue was purified by column chromatography using a gradient mixture of hexane/ethyl acetate as the eluent, yielding 14.31 g of a yellowish viscous liquid (86 % yield). In the third stage, AECA was hydrolyzed to obtain ACA. Specifically, 27.51 g (118 mmol) of AECA, 100 mL of distilled water and 4.74 g (118 mmol) of sodium hydroxide were added to a 100 mL three-necked round-bottom flask equipped with a condenser, thermometer and magnetic stirrer. The resultant mixture was heated to reflux for 4 h. After cooling, the mixture was acidified to pH 3 with concentrated hydrochloric acid. The resulting solid was filtered off and dried in a vacuum oven at 50 °C for 24 h. This process yielded 22.72 g of the desired product as a white solid with a yield of 94%.

### 2.3. Synthesis of 2-((2-(3-((2-Aminoethyl)thio)4-methylcyclohexyl)propyl)thio)ethanamine (LC)

The synthesis of this compound was performed according to a method previously reported by our research group [37]. Typically, 10 g (0.65 moles) of limonene, 44.78 g cysteamine hydrochloride (0.39 moles), 1.38 g (3.2 × 10^−3^ moles) of BAPO and 150 mL of absolute ethanol were charged into a 250 mL Erlenmeyer flask with a glass stopper. The resulting solution was irradiated with a Bluewave 200 Dymax UV-Vis lamp (Torrington, CT, USA) using a UV light intensity of 150 mW/cm^2^ for 8 h. After this time, the solvent of the reaction mixture was roto-evaporated and the residue was rinsed with hexane and subsequently re-dissolved in 50 mL of chloroform. This organic solution was then extracted with an aqueous solution of sodium hydroxide 10% *w*/*v* (4 × 30 mL) to remove the excess of the cysteamine hydrochloride. The desired product was obtained in a quantitative yield as a clear yellow dense liquid.

### 2.4. Synthesis of (E)-3-(4-(Allyloxy)phenyl)-N-(2-((2-(3-((2-((E)-3-(4-(allyloxy)phenyl)acrylamido)ethyl)thio)-4-methylcyclohexyl)propyl)thio)ethyl) Acrylamide (LCA)

In a 250 mL three-necked round-bottom flask equipped with a condenser, magnetic stirrer, thermometer and a Dean-Stark trap, 8.64 g (42 mmol) of ACA, 33 mg (0.53 mmol) of dried boric acid and 110 mL of toluene were charged. Next, 3.0 g (11 mmol) of LC was added and the resulting reaction mixture was refluxed until the calculated amount of water was collected in the trap. Then, the mixture was allowed to cool and filtered to remove the catalyst. The filtrate was poured into 500 mL of hexane and the precipitated solid was filtered and rinsed with 60 mL of hexane, followed by washings with distilled water (3 × 60 mL). The obtained solid was vacuum dried for 12 h and subsequently purified by column chromatography using a gradient mixture of hexane/ethyl acetate as the eluent. This process yielded 3.13 g (45% yield) of a white solid with an m.p. of 100 °C. ^1^H NMR (500 MHz, CDCl_3_) δ (ppm): 7.59 (d, *J* = 15.50 Hz, 2H, -NH-C=O-CH=CH-Ar), 7.44 (d, *J* = 6.64 Hz 4H, *o*-Ar-H), 6.88 (dd, *J* = 3.60 Hz, 4H, *m*-Ar-H), 6.34 (dd, *J* = 3.04 Hz 4H, -NHC=O-CH=CH-Ar + NH-overlapped), 6.05 (m, 2H, -O-CH_2_-CH=CH_2_), 5.42 (d, *J_1_* = 16.88 Hz, 2H, -O-CH_2_-CH=CH_2_), 5.31 (d, *J_2_* = 10.30 Hz, 4H, -O-CH_2_-CH=CH_2_), 4.55 (t, *J* = 4 Hz 4H, -O-CH_2_-CH=CH_2_), 3.57 (m, 4H, -S-CH_2_-CH_2_-NH), 2.72 (m, 4H, , -S-CH_2_-CH_2_-NH), 2.63 (m, 1H, -CH-S-CH_2_-CH_2_-NH), 2.34 (m, 2H, -CH_2_-S-CH_2_-CH_2_-NH), 2.0–1.5 (m, 9H, cycloaliphatic protons), 1.02 (d, *J* = 6.58 Hz, 3H, -CH_3_) and 0.96 (dd, *J* = 6.85, 3H, -CH_3_). FTIR in KBr, cm^−1^: 3272 stretch NH-CO; 3076 stretch C-H Ar; 2921 stretch Aliph C-H; 1653 stretch -C=O-NH; 1602 Ar -C-H; 1540 stretch C-N; 1222 stretch C-O; 928 stretch -C=C-.

### 2.5. Synthesis of (E)-3-(4-(Allyloxy)-3-methoxyphenyl)-N-(2-((2-(3-((2-((E)-3-(4-(allyloxy)-3-methoxyphenyl)acrylamido)ethyl)thio)-4-methylcyclohexyl)propyl)thio)ethyl) Acrylamide (LFA)

For this, 6.55 g (28 mmol) of AFA, 28 mg (0.47 mmol) of dried boric acid, 2.71 g (9 mmol) of LC and 110 mL of toluene were charged into a 250 mL three-necked round-bottom flask equipped with a condenser, magnetic stirrer, thermometer and a Dean–Stark trap. The resulting reaction mixture was refluxed until the calculated amount of water was collected in the trap. Then, the mixture was allowed to cool and filtered to remove the catalyst. The filtrate was poured into 500 mL of hexane and the precipitated solid was filtered and rinsed with 60 mL of hexane, followed by washings with distilled water (3 × 60 mL). Then, the obtained solid was vacuum-dried for 12 h and subsequently purified by column chromatography using a gradient mixture of hexane/ethyl acetate as the eluent. This process yielded 2.78 g (42% yield) of a white solid with an m.p. of 60 °C. ^1^H NMR (500 MHz, CDCl_3_) δ (ppm): 7.56 (d, *J* = 15.48 Hz, 2H, -NH-C=O-CH=CH-Ar), 7.04 (m, 4H, Ar-H), 6.82 (m, 2H, Ar-H), 6.35 (m, 4H, -NHC=O-CH = CH-Ar + NH-overlapped), 6.05 (m, 2H, -O-CH_2_-CH=CH_2_), 5.41 (d, *J* = 17.17, -O-CH_2_-CH=CH_2_), 5.30 (d, *J* = 10.50, -O-CH_2_-CH=CH_2_), 4.62 (s, 4H, -O-CH_2_-CH=CH_2_), 3.87 (s, 3H, -OCH_3_), 3.57 (m, 4H, -S-CH_2_-CH_2_-NH), 2.72 (m, 4H, -S-CH_2_-CH_2_-NH), 2.61(m, 2H, -CH-S-CH_2_-CH_2_-NH), 2.42 (m, 2H, -CH_2_-S-CH_2_-CH_2_-NH), 2.30–1.20 (m, 9H, cycloaliphatic protons), 1.01 (d, *J* = 6.68 Hz, 3H, -CH_3_) and 0.95 (dd, *J* = 7.18, 3H, -CH_3_). FTIR in KBr, cm^−1^: 3284 stretch NH-CO; 3078 stretch C-H Ar: 2925 stretch Aliph C-H; 1657 stretch -C=O-NH; 1600 Ar -C-H; 1509, C-N; 1255 stretch C-O: 929 stretch -C=C.

### 2.6. 3-(4-(Allyloxy)phenyl)-N-(2-((2-(3-((2-(3-(4-(allyloxy)phenyl)propanamido)ethyl)thio)-4-methylcyclohexyl)propyl)thio)ethyl) Propanamide (LPA)

In a 250 mL three-necked round-bottom flask equipped with condenser, magnetic stirrer, thermometer and a Dean–Stark trap, 5.71 g (27 mmol) of APA, 85 mg (1.4 mmol) of dried boric acid and 110 mL of toluene were charged. Next, 4.0 g (14 mmol) of LC was added and the resulting reaction mixture was refluxed until the calculated amount of water was collected in the trap. Then, the mixture was allowed to cool and filtered to remove the catalyst. The filtrate was poured into 500 mL of hexane and the produced precipitated solid was filtered and rinsed with 60 mL of hexane, followed by washings with distilled water (3 × 60 mL). The obtained solid was vacuum-dried for 12 h and subsequently purified by column chromatography using a gradient mixture of hexane/ethyl acetate as the eluent. This process yielded 3.33 g (36% yield) of a white solid with an m.p. of 90 °C. ^1^H NMR (500 MHz, CDCl_3_) δ (ppm): 7.11 (d, *J* = 8.52 Hz, 4H, *o*-Ar-H), 6.84 (d, *J* = 8.62 Hz, 4H, *m*-Ar), 6.06 (m, 2H, -O-CH_2_-CH = CH_2_), 5.91 (m, 2H, NH-C=O), 5.41 (dq, *J* = 1.51 Hz, 4H, -O-CH_2_-CH=CH_2_ ), 5.29 (dq, *J* = 1.3 Hz, 4H, -O-CH_2_-CH=CH_2_ ), 4.52 (m, 4H, -O-CH_2_-CH=CH_2_, 3.40 (m, 4H, -S-CH_2_-CH_2_-NH), 2.91 (t, *J* = 7.55 Hz, 2H, NH-C=O-CH_2_-CH_2_-Ar), 2.59 (m, 4H, -S-CH_2_-CH_2_-NH), 2.46 (t, *J* = 7.87 Hz, 2H, NH-C=O-CH_2_-CH_2_-Ar), 2.36 (m, 1H, -CH-S-CH_2_-CH_2_-NH), 2.24 (m, 2H, -CH_2_-S-CH_2_-CH_2_-NH), 2.06–1.03 (m, 9H, cycloaliphatic protons), 1.00 (d, *J* = 6.92 3H, -CH_3_) and 0.94 (dd, *J* = 3.07 Hz, 3H, -CH_3_). FTIR in KBr, cm^−1^: 3303 stretch NH-CO; 3084, stretch C-H Ar; 2922 stretch Aliph C-H; 1637 stretch -C=O-NH; 1613 Ar -C-H; 1510, stretch C-N: 1240, stretch C-O, 923 stretch -C=C.

### 2.7. Synthesis of 2,3-Bis(3-Sulfanylpropanoyloxy)propyl 3-sulfanylpropanoate (GTMP)

The bio-based trifunctional thiol derived from the esterification of glycerol with 3-mercaptopropionic acid was prepared according to a recent method reported by our research group [38]. Typically, in a 100 mL three-necked round-bottom flask provided with a thermometer, magnetic stirrer and a condenser fitted with a Dean–Stark trap, 75 mL of dry toluene, 4.50 g (48.8 mmol) of glycerol, 18.67 g (175.9 mmol) of 3-MPA and 0.348 g (1.83 mmol) of p-TSA were charged. This reaction mixture was stirred and heated to 90 °C for six hours until the collection of a stoichiometric amount of water was collected in the Dean–Stark trap. Next, the reaction mixture was washed with 100 mL of a NaHCO_3_ solution (10 wt.%) and a saturated aqueous solution of NaCl (150 mL, three times). Then, the organic phase was collected, dried over Na_2_SO_4_, filtered and rotary evaporated to yield a colorless viscous liquid. The trifunctional thiol was isolated by column chromatography using a gradient mixture of hexane/ethyl acetate as the eluent and analyzed. The glycerol-derived trifunctional thiol GTMP was obtained as a colorless, odorless and slightly viscous liquid (7.1 g, 40%).

### 2.8. Bulk Photopolymerization of Photocurable Formulations

Table 1 illustrates the amounts in grams and moles of the components in the photocurable formulations involving the prepared monomers such as LCA, LFA and LPA along with the bio-based trifunctional thiol GTMP and the photoinitiator DMPA. The formulations were designed to use monomer-to-thiol ratios of 1.5:1 and 1:1.33. In the former formulation, it was considered that the trifunctional thiol only reacts with two allylic double bonds, while in the latter, the thiol can react with two allylic and two enone double bonds.

Each monomer was heated until it melted using a heat gun and once melted it was poured into a metallic mold. Then, the thiol GTMP was added together with the photoinitiator DMPA and the resulting solution was homogenized. The mold was introduced in a dark chamber provided with a Bluewave Dymax ultraviolet spot-lamp (Torrington, CT, USA) coupled with a fiber optic guide. The output of the lamp was set to an intensity of 100 mW/cm^2^, using a control Cure radiometer UVProcessSupply, (Chicago, IL, USA). The formulations were irradiated for 30 min and then the resulting polymers were demolded and further analyzed by calorimetric techniques.

### 2.9. Characterization of Samples

NMR analyses were carried out on a Bruker Fourier Ultrashield 500 MHz NMR spectrometer (Billerica, MA, USA) using deuterochloroform and dimethyl sulfoxide-d6 as the solvents and tetramethylsilane as the reference. Melting points (m.p.) were determined in a Fisher-Johns melting point apparatus. FTIR spectroscopy analyses were performed in a Nicolet 6700 FTIR spectrometer (ThermoFisher Scientific, Shirley, NY, USA) using dry KBr to suspend the sample. The MALDI-TOF analysis of the synthesized monomers was performed using an Autoflex Speed Bruker MALDI-TOF instrument, Billerica, MA, USA. A DCTB matrix (2,5-Dihydroxybenzoic acid (DHB) and 2-Cyano-4-hydroxycinnamic acid (CHCA) mixed matrix) was used and the method employed was a positive reflection at a 28% laser intensity. UV analysis was accomplished on a Horiba Duetta Absorbance spectrometer (Kyoto, Japan), using HPLC chloroform (Sigma-Aldrich, Toluca, Mexico) as the solvent. A dynamic mechanical analysis (DMA) was performed using a TA Instruments Q800 DMA (New Castle, DE, USA). The test specimens were obtained using a steel mold with dimensions of 40 mm long, 10 mm wide and 2 mm thick. The measurements were performed in tension mode, heating the samples from 30 to 130 °C at 5 °C min^−1^ and a frequency of 1 Hz. The temperature at the maximum in the Tan δ curve was taken as the glass transition temperature, T_g_. The cross-link density (*ρ*) was calculated according to the rubber elasticity theory using Equation (1):

(1) $$\rho =\frac{G^{\prime }}{3RT}$$

where *G*′ is the storage modulus in the rubbery region (at T_g_ + 50 °C), *R* is the universal gas constant (8.314 J/(mol∙K)) and *T* is the absolute temperature in Kelvin degrees (K).

The stress–strain curves were obtained using the DMA technique using a tensile clamp configuration. The samples were stretched at a controlled frequency and temperature. The experiments were conducted isothermally at 30 °C with a strain rate ramp of 0.02 mm/min, ranging from 0.0 to 20 mm. The elastic modulus (E) was determined from the slope of the initial linear region of the stress–strain (σ-ε) curve.

DSC measurements were performed using a TA Instruments Discovery 2500 differential scanning calorimeter (New Castle, DE, USA). The equipment was set to heat the samples from −50 °C to 280 °C at a heating rate of 5 °C/min in a N_2_ atmosphere. The T_g_ was determined from the second heating curve. The TGA analysis was performed using a TA Instruments TGA Q500 (New Castle, DE, USA). The analysis was conducted from 30 °C to 600 °C at a heating rate of 10 °C/min under a N_2_ atmosphere.

## 3. Results

### 3.1. Synthesis of the Bio-based Monomers

**Scheme 2:**
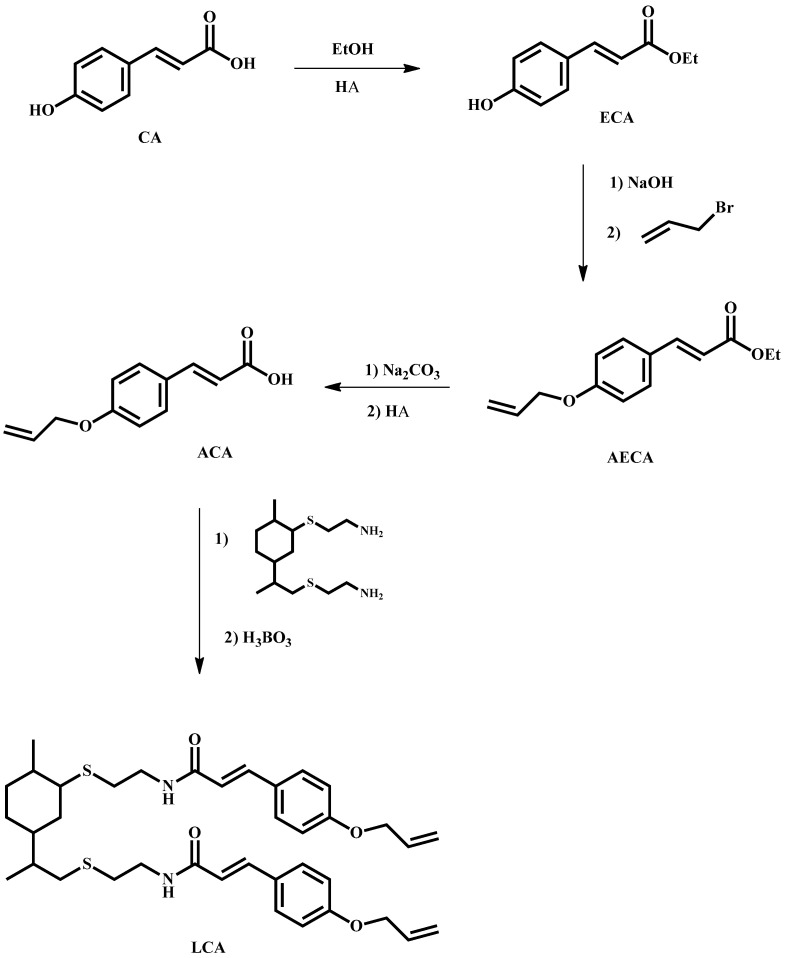
Synthetic methodology to prepare the monomer LCA.

The hybrid bio-based aromatic–aliphatic monomers were synthesized through a three-stage procedure (see Scheme 2). In the initial stage, allylated derivatives of coumaric acid, ferulic acid and phloretic acid were synthesized in three steps: (1) protecting the carboxylic group via esterification with ethanol, (2) alkylating with allyl bromide using the Williamson etherification reaction and (3) hydrolyzing the allylated esters under basic conditions followed by acidification to pH 3 using hydrochloric acid. Moving to the second stage, we prepared a bio-based diamine compound (LC) by employing a photo-induced thiol-ene coupling reaction between limonene and cysteamine. Finally, in the third stage, the allylated acids and LC were condensed in the presence of boric acid as the catalyst to obtain the corresponding diamides denoted as LCA, LFA and LPA.

**Figure 1:**
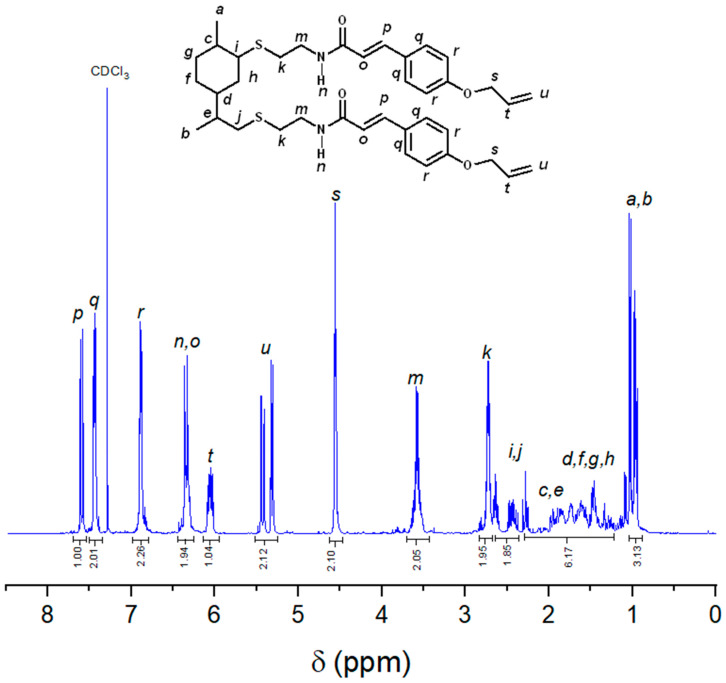
^1^H NMR spectra of LCA run in CDCl_3_.

Figure 1 illustrates the ^1^H NMR spectrum of LCA. The aromatic protons corresponding to the coumaric acid moiety appear as two signals at 7.44 (*q*) and 6.88 (*r*) ppm, each integrating for two protons. The double bond protons are observed as two doublets, one at 7.59 ppm (*p*) and the other at 6.34 ppm (*o*). The amide group protons (*n*) overlap with one of the double-bond peaks (6.34 ppm), resulting in a signal that integrates for two protons. The allyl group protons (*t*, *u*, *s*) exhibit three sets of signals in the range of 6.2 to 4.56 ppm with an integration ratio of 1:2:2. The methylene protons adjacent to the amide group (*m*) were found as a multiplet at 3.58 ppm, while those connected to the sulfur atom (*k*, *i*, *j*) are located in the range of 2.72 to 2.48 ppm. The protons from the cyclohexane ring (*c*–*h*) appear as multiplet signals in the range of 2.05 to 1.05 ppm, while the methyl groups exhibit a doublet (*a*) at 1.02 ppm and a doublet of doublets (*b*) at 0.95 ppm. Additionally, a ^1^H NMR COSY study of LCA (refer to Figure S1) reveals coupling between the amide group protons and the protons of the adjacent carbons on both sides of this moiety.

**Figure 2:**
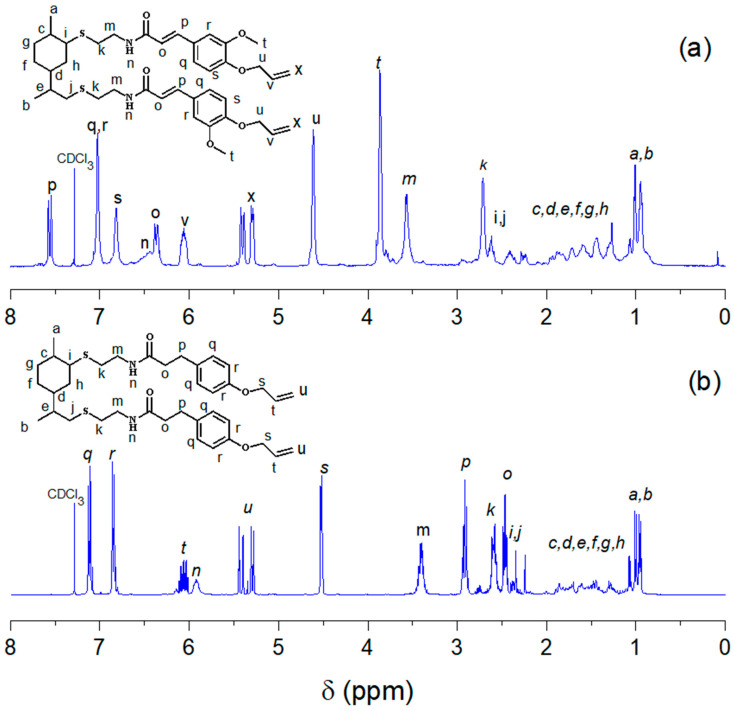
Comparison of the ^1^H NMR spectra of (**a**) LFA and (**b**) LPA, run in CDCl_3_.

The three monomers under investigation exhibit structural analogies, leading to similar ^1^H NMR spectra with subtle variations. In Figure 2 is depicted the comparison of the ^1^H NMR spectra between LFA and LPA. The spectrum of LFA reveals a distinct pattern of signals for the aromatic protons due to the substituent in the meta position, as well as the presence of the peak corresponding to the methoxy group (*t*) at 3.87 ppm. In the case of the spectrum of LPA, the proton of the amide group (*n*) is significantly shifted to a higher field (5.93 ppm) compared to LFA (6.44 ppm) due to the absence of the double bond that has an inductive effect. Additional multiplet signals are observed at 2.91 and 2.46 ppm, corresponding to the methylene protons adjacent to the amide group (*o*) and those associated with the aromatic ring (*p*).

**Figure 3:**
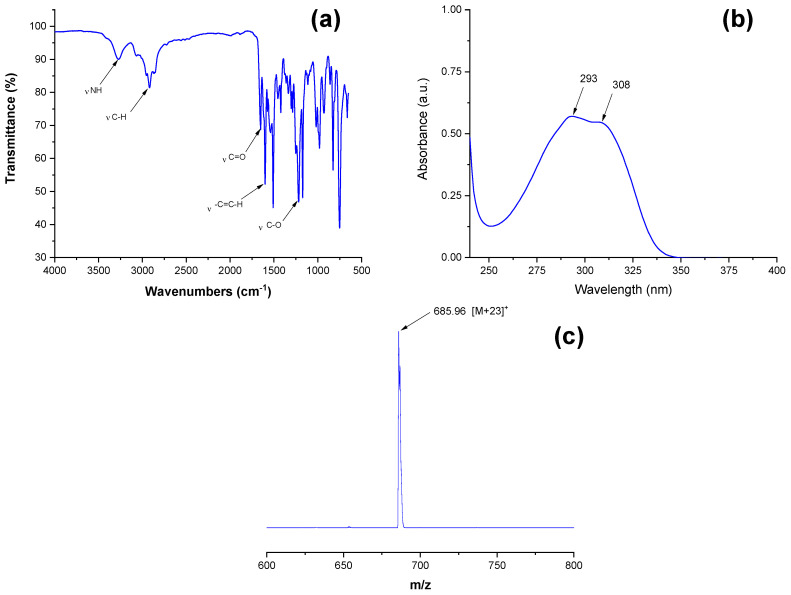
Chemical characterization of LCA: (**a**) FTIR spectrum in KBr, (**b**) UV-Vis spectrum in CHCl_3_ and (**c**) MALDI-TOF spectrum.

Figure 3 presents the FTIR spectrum, UV-Vis spectrum and mass-spectrum obtained from Matrix-Assisted Laser Desorption/ionization Time of Flight (MALDI-TOF) analysis for LCA. The FTIR spectrum of LCA exhibits several characteristic bands indicative of its chemical structure. A stretching band at 3272 cm^−1^ indicates the presence of the N-H bond in the amide group. Bands centered at 2921 cm^−1^ correspond to the stretching mode of the aliphatic C-H bonds of the cyclohexane ring. A distinct band localized at 1656 cm^−1^ is characteristic of the carbonyl group in the amide, while the band at 1602 cm^−1^ corresponds to the aromatic stretch C=C-H bonding. Additionally, a band at 926 cm^−1^ is attributed to the double bond in the allyl groups. LFA and LPA exhibit similar FTIR spectra to that of LCA as all three compound possess the same functional groups.

The UV-Vis spectrum of the monomer LCA exhibits two absorption maxima at 293 and 315 nm, consistent with values reported for other hydroxy cinnamic acid derivatives [39,40]. These maxima are attributed to the double bond conjugated with the aromatic ring and the carbonyl group of the amide. The MALDI-TOF mass spectrum displays the ion intensity versus the mass-to-charge (*m*/*z*) ratio, featuring a peak at *m*/*z* 685.96 [M+ 23]^+^. This peak matches precisely with the expected molecular weight of the compound (662.94) plus the molecular weight of a sodium ion (23). These chemical characterizations confirm the identity of the synthesized compound. Figures S2 and S3 depict the FTIR, UV and MALDI-TOF for LFA and LPA.

### 3.2. Photoisomerization and Photocycloaddition of the Enone Double Bonds of the Synthesized Monomers

The isomerization in cinnamate derivatives is a complex process driven by the absorption of UV light, which excites the electrons in the π-conjugated system from the ground state to the first excited singlet state (S1). In this excited state, the π−electrons move to an antibonding π* orbital, while their spins remain paired. This results in the weakening of the π-bond between the carbon atoms, effectively reducing the bond order from a double bond to a single bond. With the double bond weakened, the molecule gains sufficient flexibility to allow rotation around the bond. This rotational movement is relatively rapid and can lead to the interchange of substituent positions, transforming the spatial arrangement from the *E* (trans) form to the *Z* (cis) form. After rotation, the molecule can relax back to the ground state by releasing the absorbed energy, often through nonradiative decay pathways such as internal conversion or intersystem crossing to a triplet excited state. In the triplet state, the molecule may also undergo rotation around the weakened double bond before relaxing back to the ground state [41,42]. Upon returning to the ground state, the molecule stabilizes in the new isomeric form. The absorbed energy during excitation and subsequent release allows the double bond to re-form, locking the substituents in the Z (cis) configuration. This phenomenon is well-known in cinnamic acid derivatives [43,44].

The [2+2] photocycloaddition of cinnamate derivatives involves five steps, including photoexcitation (PE), intersystem crossing (ISC), the first C-C bond formation, another ISC to the singlet diradical and a direct diradical combination to form a second C-C bond, forming a cycloadduct. The cycloaddition reaction has evolved from the early discoveries, trying to understand the basic principles of photochemistry and the behavior of the excited states, to the modern computerized mechanistic studies, in which parameters such as the use of a catalyst, solvents and light sources have been optimized to achieve higher yields and better selectivity. There have been significant advances in the integration of new technologies, such as high-throughput screening, computational modeling and advanced spectroscopic techniques to better understand and control photocycloaddition reactions [45]. The [2+2] photocycloaddition reactions have a wide range of applications, for instance, in synthetic chemistry, in the development of luminescent materials and polymers through controlled cycloaddition reactions [46], and in the synthesis of bioactive compounds with potential therapeutic applications [47]. Moreover, photocycloaddition reactions can be used for surface patterning, allowing for the creation of micro- and nano-scale patterns on polymer surfaces [48]. The development of biocompatible polymers through photocycloaddition can aid in tissue engineering, providing scaffolds that support cell growth and tissue regeneration [49]. Photocycloaddition reactions are also used in the fabrication of optoelectronic devices, such as organic light-emitting diodes (OLEDs) and photovoltaic cells [50]. These reactions help create materials with specific electronic properties. These kinds of reactions can help the development of smart materials that respond to external stimuli, such as light, temperature or pH, which can be achieved through photocycloaddition reactions. These materials have applications in sensors, actuators and responsive coatings [51].

**Figure 4:**
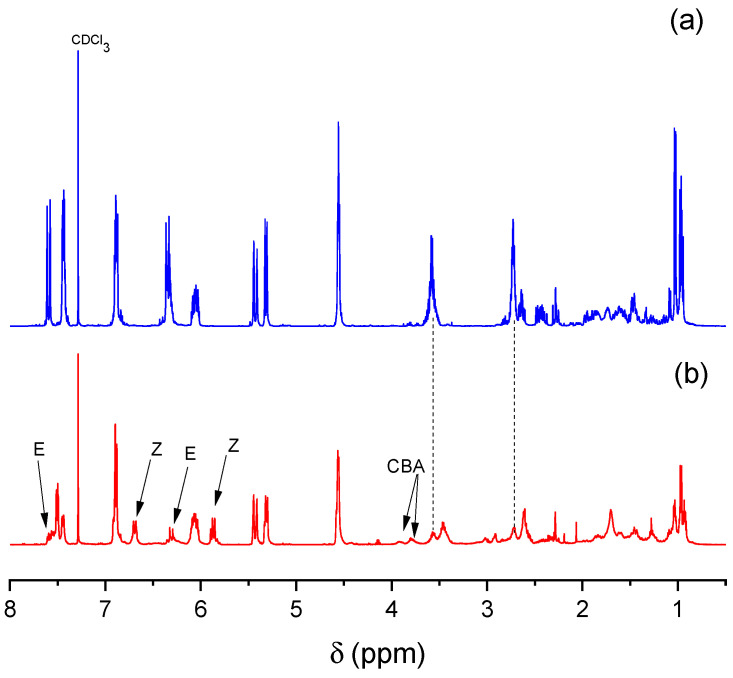
Comparison of ^1^H NMR spectra of (**a**) pristine LCA and (**b**) isomerized LCA after 1 h of UV irradiation.

In our study, we investigated the photoisomerization of the prepared monomers LCA and LFA which contain the conjugated double bond. These monomers were separately irradiated with UV light using CDCl_3_ as the solvent and chemical changes were recorded at different time intervals. A comparison of the ^1^H NMR spectrum of pristine LCA with that after UV irradiation revealed the partial transformation of the trans isomer to the cis isomer (see Figure 4). The appearance of two new olefinic proton signals (Z) at 5.86 and 6.67 ppm with a coupling constant of 12.9 Hz was observed (Figure 4b). Simultaneously, the signals corresponding to the doublets of the trans isomer (*E*) at 7.58 and 6.30 ppm, with a coupling constant of 15.4 Hz, decreased. The aromatic protons in the ortho position also underwent significant changes due to photoisomerization. The original doublet of the aromatic proton at 7.43 ppm in LCA transformed into a doublet of doublets in the isomerized form. This transformation arises from the long-range coupling of the proton of the double bond in the cis position with the proton in the ortho position of the aromatic ring. Analogous changes after UV irradiation were observed for the monomer LFA, though in this case, a more rapid isomerization of the double bond was found.

The results of our ^1^H NMR study on the photoisomerization and photocycloaddition reactions of the prepared cinnamate derivatives are consistent with other related reports. For example, Kort et al. [52] reported a ^1^H NMR study of the photoisomerization of the p-coumaric. They observed the appearance of new signals at 6.28 and 5.76 ppm, corresponding to the protons of the double bonds in the cis isomer of p-coumaric acid, in addition to those of the double bonds of the trans isomer. The coupling constants of 15.9 Hz for the trans isomer and 12.8 Hz for the cis isomer also agree with the values obtained in our study (15.4 and 12.9 Hz). Additionally, Danylec and Iskander [53] reported the ^1^NMR study on the partial conversion of methyl p-hydroxy-trans-cinnamate to the thermodynamically less stable cis isomer, indicated by the appearance of the cis olefinic proton at 5.78 ppm. Similar results were reported by Park et al. [54] who observed the appearance of peaks at 5.9 and 7.2 ppm corresponding to the protons of the double bond of the cis isomer.

Regarding the [2+2] photocycloaddition reaction of the prepared monomers, it was found that this reaction proceeded simultaneously with the isomerization reaction, as evidenced by the appearance of new signals at 3.78 and 3.91 ppm (see Figure 4b). Photocycloaddition reactions involving the double bond of cinnamic acid derivatives are also common in these compounds [55]. These reactions can occur either intramolecularly or intermolecularly, leading to the formation of cyclobutane adducts (CBA). The resulting adducts can be labeled as head-to-head (HH), when substituents are on the same side (1,2-relationship), or head-to-tail (HT), when substituents are on opposite sides (1,3-relationship). Additionally, syn and anti isomers can form due to the spatial configuration of the substituents in the cyclobutane ring. In the syn isomer, identical groups are on the same face (top or bottom) regardless of their location (1,2 or 1,3). In contrast, the anti isomer has identical groups on opposite faces. Consequently, the cycloaddition reaction can theoretically yield more than 10 distinct isomers [56].

**Figure 5:**
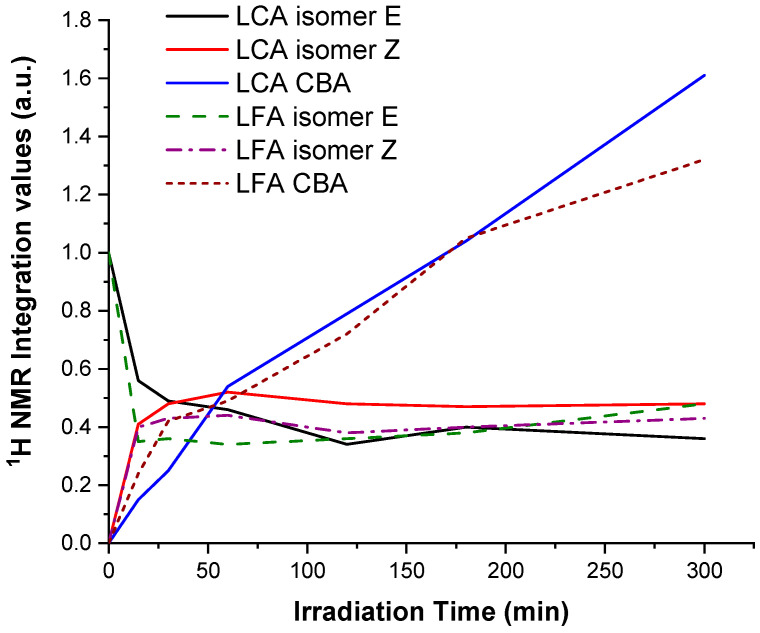
^1^H NMR study of the photo-isomerization and photocycloaddition reactions involving the integration of the peaks at 6.30 (E), 5.86 (Z) and 3.57 ppm (CBA) in the spectra of LCA and LFA.

To monitor the progression of photoisomerization and photocycloaddition reactions in LCA and LFA during UV irradiation, we integrated the signals at 6.30, 5.86 and 3.57 ppm at various time intervals. The proton of the double bond in the allyl group at 6.05 ppm remained constant throughout the irradiation period and was thus used as a reference, with its integration set to 1. This reference allowed for the determination of the relative amounts of the cis and trans isomers, as well as the cycloaddition adducts (CBA). The proton of the isomer E in both monomers at 6.30 ppm initially displayed an integration value of 1. Figure 5 depicts the evolution of these species for both LCA and LFA.

A similar behavior was observed for the photoisomerization reaction of both monomers. There was a rapid decline in the integration of the proton of the E isomer within the first 30 min, followed by a plateau after 60 min, achieving final values of 0.38 and 0.36 for LCA and LFA, respectively, after 300 min. However, a more rapid transformation of isomer E to isomer Z was observed for LFA in the initial minutes of irradiation. This could be attributed to the electronic effect of the methoxy group in LFA, that may enhance the efficiency of the intersystem crossing, leading to faster isomerization compared to LCA. Additionally, the photophysical properties, such as the absorption spectra and quantum yield, can differ between ferulic and coumaric acid derivatives. The ferulic acid derivative may have a higher quantum yield for isomerization, contributing to slightly faster isomerization rates. The formation of the isomer *Z* in both monomers also displayed a similar trend: increasing values in the first 30 min, achieving values of 0.48 and 0.42 for LCA and LFA, respectively, followed by a plateau at these values for both monomers.

Regarding the photocycloaddition reaction, it proceeded in both cases from the onset of UV irradiation, though more rapidly in the case of LFA. Final values of 1.61 and 1.32 were achieved for LCA and LFA, respectively, after 300 min. This indicates that the photoisomerization and photodimerization reactions proceed simultaneously during the irradiation period. The photocycloaddition [2+2] can proceed either intra- or intermolecularly, producing a series of isomers.

**Scheme 3:**
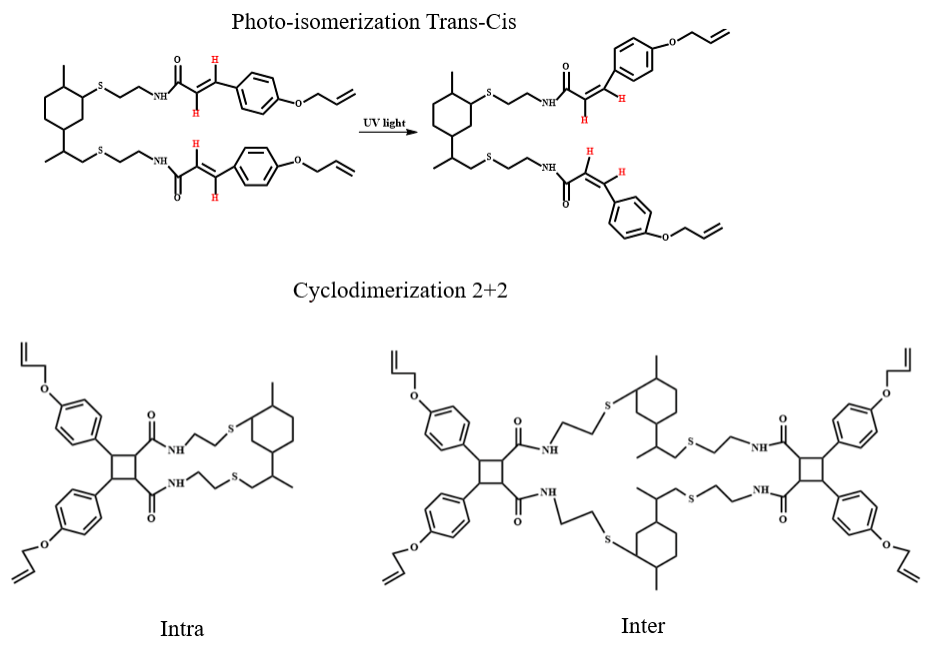
Proposed chemical structures of the compounds resulting from the photoisomerization and photocycloaddition 2+2 reactions of LCA.

Scheme 3 provides a simplified representation of the potential products formed during both photoisomerization and photocycloaddition reactions.

The isomerization reaction in cinnamate derivatives is generally faster than (2+2) cycloaddition reactions due to several key factors. First, isomerization involves the rotation around a double bond, which typically requires less activation energy compared to the formation of new bonds in the cycloaddition reactions. Cycloaddition reactions necessitate a higher energy input to proceed. Moreover, in the excited state, the π–electrons of the cinnamate derivatives are promoted to an antibonding orbital, weakening the double bond, allowing easier rotation. This rotation can occur rapidly without significant structural rearrangements. Additionally, the excited state of the bio-based monomers can undergo non-radiative decay pathways, such as internal conversion or intersystem crossing, which facilitate the isomerization process. These pathways are generally more efficient for isomerization than for cycloaddition reactions. Finally, steric hindrance in the cycloaddition reactions can slow down the process, as the approach of two double bonds to form a cyclobutane ring requires precise alignment. In contrast, the isomerization reaction involves the rotation of substituents around a double bond, which is less sterically demanding. These factors contribute to the faster rate of double bond isomerization in the bio-based monomers compared to cycloaddition reactions.

### 3.3. Thiol-Ene Photopolymerization

In this study, we employed the synthesized hybrid aliphatic–aromatic monomers in combination with the trifunctional thiol GTMP to produce fully bio-based poly(amide–thioether)s via the thiol-ene photopolymerization process. The formulations were prepared utilizing monomer-to-thiol ratios of 1.5:1 and 1:1.33. At the 1.5:1 ratio, only the allylic double bond engages in the reaction with the thiol, whereas at the 1:1.33 ratio, the excess of the thiol could react with both the allylic and enone double bonds. Due to the solid state of all three monomers, we first melted them in a metallic mold before combining them with the thiol and photoinitiator to prepare the photocurable formulations.

**Figure 6:**
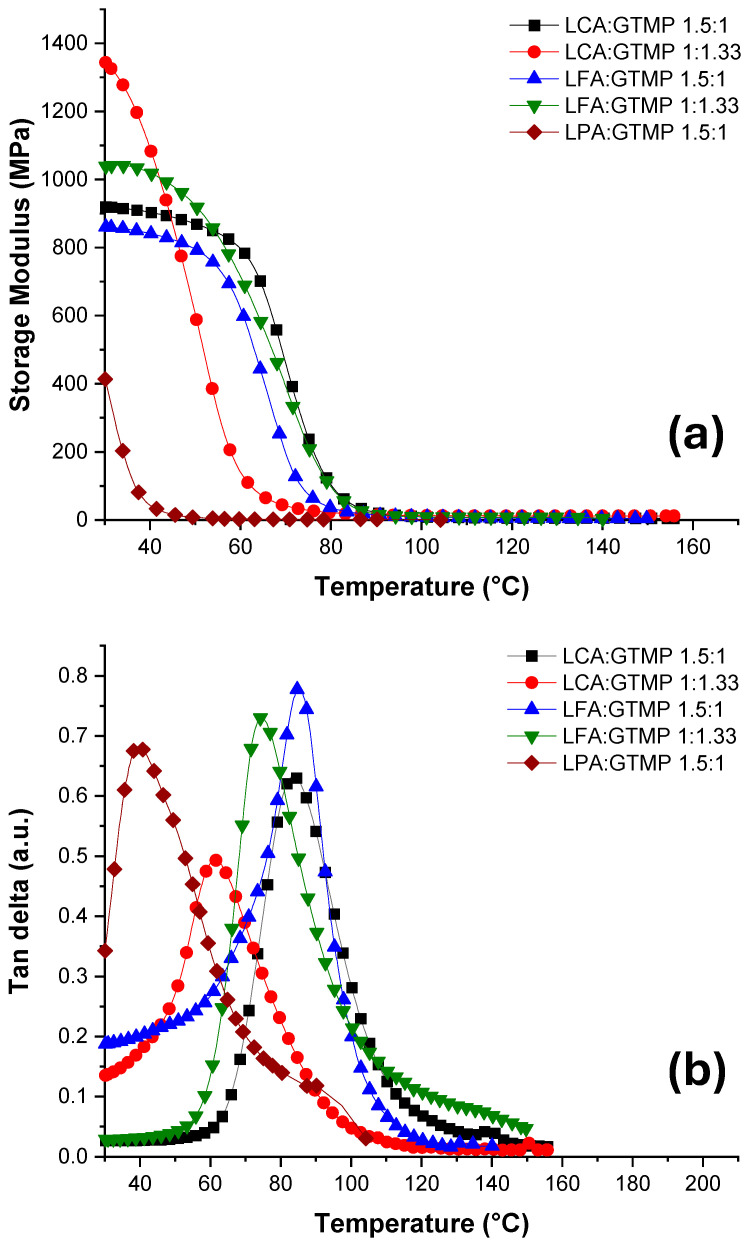
DMA results of the cured test specimens derived from the hybrid monomers prepared: (**a**) storage modulus of poly(amide-thioethers) derived from formulations LCA, LFA and LPA with GTMP at 1.5:1 and 1:1.33 ratios; (**b**) Tan delta curves for polymers derived from the same formulations.

The formulations were subjected to non-filtered UV-Vis radiation to synthesize the corresponding poly(amide–thioether)s. The resultant polymers were subsequently analyzed using Dynamic Mechanical Analysis (DMA) to assess their viscoelastic properties. Figure 6 illustrates the storage moduli and tan delta curves for the polymers.

It has been generally observed that polymers derived from cinnamic acid derivatives exhibit rigidity attributed to the aromatic α−β enone conjugated system, a finding corroborated by our study. The variations in the chemical structures of LCA, LFA and LPA, along with the monomer-to-thiol ratio, had a significant influence on the thermal and mechanical properties of the synthesized polymers. A summary of the obtained DMA results is presented in Table 2.

The poly(amide–thioether)s produced from both LCA and LFA exhibited a consistent trend when reacted with GTMP at both 1.5:1 and 1:1.33 ratios. The storage modulus recorded for LCA at the 1.5:1 ratio was 920 MPa with a T_g_ of 84 °C, while LFA exhibited a modulus of 860 MPa and a T_g_ of 85 °C. When samples with the 1:1.33 ratio were examined, higher moduli of 1344 MPa and 1037 MPa with T_g_ values of 62 °C and 75 °C for LCA and LFA, respectively, were achieved. This increase in the modulus for both types of polymers is attributed to the increased crosslink density achieved when both types of double bonds react with the thiol, as discussed below. In contrast, the poly(amide–thioether)s obtained from LPA demonstrated a relatively low modulus of 414 MPa, attributed to the absence of an enone double bond, which results in more flexible materials. The T_g_ for this polymer was determined to be 39 °C. The observed decrease in T_g_ for polymers derived from the formulations at 1:1.33 ratios can be attributed to a higher concentration of flexible thioether groups within the polymeric matrix.

The higher T_g_ values of the prepared polymers, compared to those reported for bio-based polymers derived from thiol-ene photopolymerizations [32,33,34,35,36,57,58,59], are likely attributed to the presence of an aromatic conjugated system from the cinnamic moieties and the rigid structure associated with the cycloaliphatic ring of the limonene unit. Additionally, the presence of amide groups in the crosslinked network facilitates the formation of hydrogen bonding, leading to a more compact packing with increased rigidity [60]. This finding is particularly significant as polythioethers synthesized through thiol-ene photopolymerization generally exhibit low T_g_ and mechanical properties due to their inherent flexibility.

For formulations with a 1:1.33 ratio involving LCA and LFA, the increased availability of thiol allows concurrent reactions with both types of double bonds, leading to higher crosslink densities of 1294 mol/m^3^ and 792.77 mol/m^3^, respectively, compared to 624 and 663 mol/m^3^ observed for the 1.5:1 ratio. The poly(amide–thioether) derived from LPA exhibited a crosslink density of 221 mol/m^3^. Theoretically, in the 1.5:1 formulation, the terminal allylic double bond is expected to preferentially react with the thiol as internal double bonds typically react more slowly owing to steric hindrance from substituents [27]. Conversely, in the 1:1.33 scenario, provided a sufficient reaction time is allowed, thiols are anticipated to effectively react with both types of double bonds. This was confirmed by analyzing the amount of the crosslinked polymer (gel) produced during photopolymerization. For LCA and LFA at both thiol concentrations, the gel percentage was higher than 98%, indicating an almost quantitative reaction between the thiols and the double bonds. For the polymer derived from LPA, the gel percentage was 90%. It is likely that the photocycloaddition reactions in LCA and LFA play a role during thiol-ene photopolymerization, resulting in a higher crosslink density and, consequently, a higher gel content. This could explain the lower gel concentration observed in LPA.

**Figure 7:**
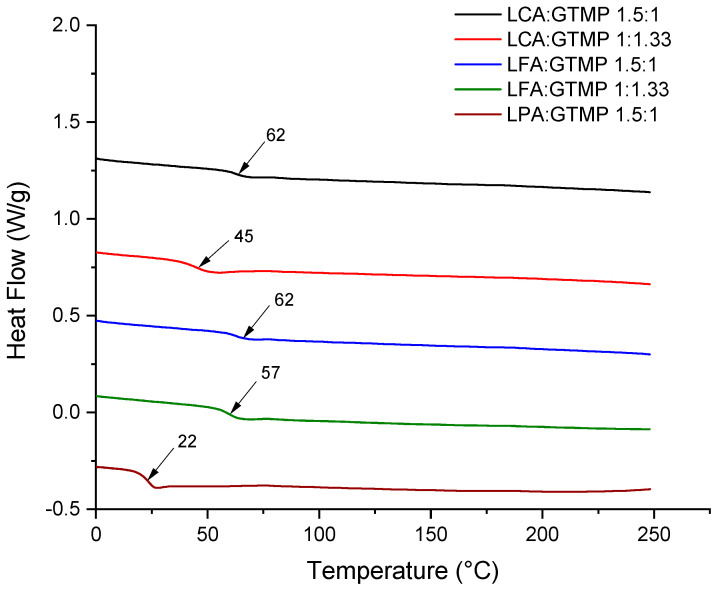
Comparison of the DSC thermograms of the obtained crosslinked poly(amide–thioether)s.

Figure 7 shows the DSC thermograms of the prepared polymers. The T_g_ obtained using DSC for the prepared polymers was consistently around 15–20 °C lower than those obtained by DMA. This discrepancy arises from the differences in how these techniques measure the glass transition. DSC measures the heat flow associated with the glass transition, detecting changes in the heat capacity of the polymer as it transitions from a glassy to a rubbery state. This method is less sensitive to subtle molecular motions and typically detects the onset of the glass transition. Additionally, DSC operates at a relatively low heating rate, corresponding to a lower frequency of molecular motions, resulting in the detection of the glass transition at a lower temperature. In contrast, DMA is more sensitive to molecular motions and can detect the full range of the glass transition, often identifying the peak of the transition. DMA involves higher frequencies due to the oscillatory nature of the applied stress or strain. Higher frequencies correspond to higher energy states, leading to the detection of the glass transition at a higher temperature. Therefore, the T_g_ obtained by DSC is generally lower because DSC is less sensitive to the full range of molecular motions ,while DMA’s higher sensitivity and frequency result in a higher detected T_g_ [61,62].

The thermal stability of the prepared polymers demonstrated a consistent trend. Poly(amide–thioether)s derived from LCA at both monomer-to-thiol ratios showed an onset of degradation (temperature at 5% weight loss) at 308 °C. In contrast, the degradation onset for LFA was 295 °C and 290 °C at both thiol concentrations. The onset of degradation for LPA was 297 °C. These similar temperatures of degradation can be attributed to the analogous chemical structures of the produced poly(amide–thioether)s.

### 3.4. Tensile Properties of the Prepared Polymers

**Figure 8:**
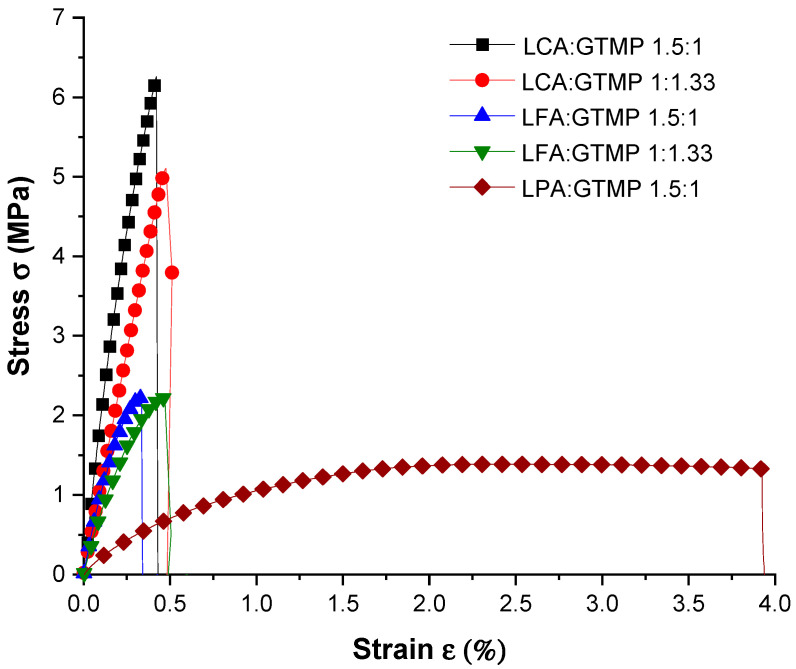
Stress vs. Strain curves for the polymers produced in this study.

The tensile mechanical properties of the synthesized polymers were evaluated, and the results are presented in Figure 8. The elastic modulus (E) was determined from the slope of the initial linear region of the stress–strain (σ-ε) curve. As previously mentioned, polymers derived from hydroxy cinnamic acid derivatives generally exhibit rigidity due to the aromatic conjugated enone system. Additionally, in our produced polymers, intermolecular hydrogen bonding between the amide groups can increase adhesion within the crosslinked network, thereby augmenting the rigidity to the polymer due to the better packing of the polymer chains.

The poly(amide–thioether)s derived from both LCA and LFA displayed a typical thermoset behavior at both polythioether contents. For LCA, E values of 15.17 and 11.02 MPa, with elongations at the break of 0.41 and 0.45%, were recorded for the polymers derived from formulations LCA:GTMP 1.5:1 and LCA:GTMP 1:1.33, respectively. The crosslinked networks derived from LFA exhibited E values of 8.49 and 6.31 MPa with elongations at the break of 0.32 and 0.45% for the 1.5:1 and 1:1.33 ratios, respectively. From these data, it can be deduced that LCA polymers are stiffer and more rigid than those derived from LFA, as denoted by the higher energy required to elongate the LCA test specimens. However, while LFA-derived polymers are stiff and rigid, they are more fragile as they break at lower stress levels. Both LCA- and LFA-derived polymers exhibited increased ductility at the higher polythioether content (1:1.33 ratio), as shown by the higher elongation at the break values.

This behavior can be attributed to the higher concentration of the flexible thioether groups in the crosslinked network, resulting in slightly more mobile networks. In the case of the crosslinked network derived from LPA, the E value was 0.88 MPa with an elongation at the break of 3.92%. This indicates higher flexibility and deformability. This behavior could be attributed to the lack of the conjugated enone system, resulting in a more flexible polymer. This observation is consistent with the DMA data, where a low storage modulus (414 MPa) and T_g_ (39 °C) were found.

## 4. Conclusions

The synthesized monomers LCA and LFA, containing enone bonds, were photochemically active, undergoing photoisomerization and photocycloaddition reactions upon UV irradiation. The resulting poly(amide–thioether)s derived from LCA and LFA, produced via thiol-ene photopolymerization, exhibiting T_g_ values in the range of 62–85 °C. This is attributed to the rigidity imparted by the aromatic α,β-unsaturated conjugated system, the cycloaliphatic ring of the limonene moiety and the hydrogen bonding between the amide groups. Consequently, these polymers demonstrated classic thermoset behavior, requiring high stress to produce minimal strain. The rigid polymers derived from LCA and LFA could be suitable for applications requiring thermal stability and enhanced mechanical properties such as in automotive, aerospace and electronics industries. Polymers with high T_g_ often exhibit increased stiffness, tensile strength and impact resistance, making them ideal for these demanding applications. In contrast, the polymer derived from LPA, despite its ability to absorb higher energy before failure, exhibited limited ductility, characteristic of brittle thermoplastics. Therefore, this polymer is more suitable for applications where moderate toughness is acceptable, but high flexibility is not critical such as rigid packaging, certain automotive parts and other applications where dimensional stability and moderate toughness are required. The prepared bio-based monomers are more sustainable and environmentally friendly compared to those derived from fossil fuels. This aligns with the growing demand for green materials in various industries, supporting the shift towards more sustainable and eco-friendly alternatives.

## Figures and Tables

**Table 1:** Components of the photocurable formulations using the prepared monomers and bio-based thiol.

Formulation	LCA		LFA		LPA		GTMP		DMPA	
	g	mol	g	mol	g	mol	g	mol	g	mol
LCA:GTMP 1.5:1.0	0.20	3 × 10^−4^	------	------	-----	------	0.07	2 × 10^−4^	3 × 10^−4^	2 × 10^−6^
LCA:GTMP 1.0:1.33	0.20	3 × 10^−4^	------	------	-----	------	0.14	4 × 10^−4^	8 × 10^−4^	3 × 10^−6^
LFA:GTMP 1.5:1.0	-----	------	0.20	3 × 10^−4^	-----	------	0.06	2 × 10^−4^	3 × 10^−4^	2 × 10^−6^
LFA:GTMP 1.0:1.33	-----	------	0.20	3 × 10^−4^	-----	------	0.13	4 × 10^−4^	8 × 10^−4^	3 × 10^−6^
LPA:GTMP 1.5:1.0	-----	------	------	------	0.20	3 × 10^−4^	0.07	2 × 10^−4^	5 × 10^−4^	2 × 10^−6^

**Table 2:** Thermal and viscoelastic properties of the polymers derived from prepared monomers.

Sample	*G*′_glassy_ (MPa)	T_g_ (DMA) (°C)	*G*′_rubbery_ (MPa)	*r* mol/m^3^	T_g_ (DSC) (°C)	Gel (%)	T_5%_ (°C)
LCA:GTMP 1.5:1	920	84	6.34	624	62	99	308
LCA:GTMP 1:1.33	1344	61	12.40	1294	45	99	308
LFA:GTMP 1.5:1	860	85	4.61	663	62	99	295
LFA:GTMP 1:1.33	1037	74	7.85	792	57	99	290
LPA:GTMP 1.5:1	414	39	2	221	22	90	297

*G*′_glassy_ determined at 30 °C, *G*′_rubbery_ determined at T_g_ + 50 °C, T_g_ considered as the maximum of tan delta curve. T_5%_ onset of degradation.

## Data Availability

Data available on request.

## Supplementary Materials

The following supporting information can be downloaded at: https://www.mdpi.com/article/10.3390/polym16233295/s1, Figure S1: COSY spectrum of LCA-run CDCl_3_; Figure S2. Chemical characterization of LFA: (a) FTIR spectrum in KBr, (b) UV-Vis spectrum in CHCl_3_ and (c) MALDI-TOF spectrum; Figure S3. Chemical characterization of LPA: (a) FTIR spectrum in KBr, (b) UV-Vis spectrum in CHCl_3_ and (c) MALDI-TOF spectrum.
