# Supplementary material for: Synthesis and Thiol-Ene Photopolymerization of Bio-Based Hybrid Aromatic–Aliphatic Monomers Derived from Limonene, Cysteamine and Hydroxycinnamic Acid Derivatives
Source: Polymers (Basel). 2024 Nov 26;16(23):3295. doi: 10.3390/polym16233295 (PMC11644351; doi:10.3390/polym16233295)
Supplement: Supplementary file 1 [file polymers-16-03295-s001.zip › polymers-3315839-supplementary.pdf]

Supplementary Material for paper "Synthesis and Thiol-Ene Photopolymerization of Biobased Hybrid Aromatic-Aliphatic monomers derived from Limonene, Cysteamine and Cinnamic Acid Derivatives.

Ricardo Acosta Ortiz*, Jorge Luis Robles Olivares, Roberto Yañez Macias.

Centro de Investigación en Química Aplicada, Department of Macromolecular Chemistry and Nanomaterials. Blvd Enrique Reyna # 140, Saltillo, Coahuila, México, 25294, Tel +528444389844 email: ricardo.acosta@ciqa.edu.mx

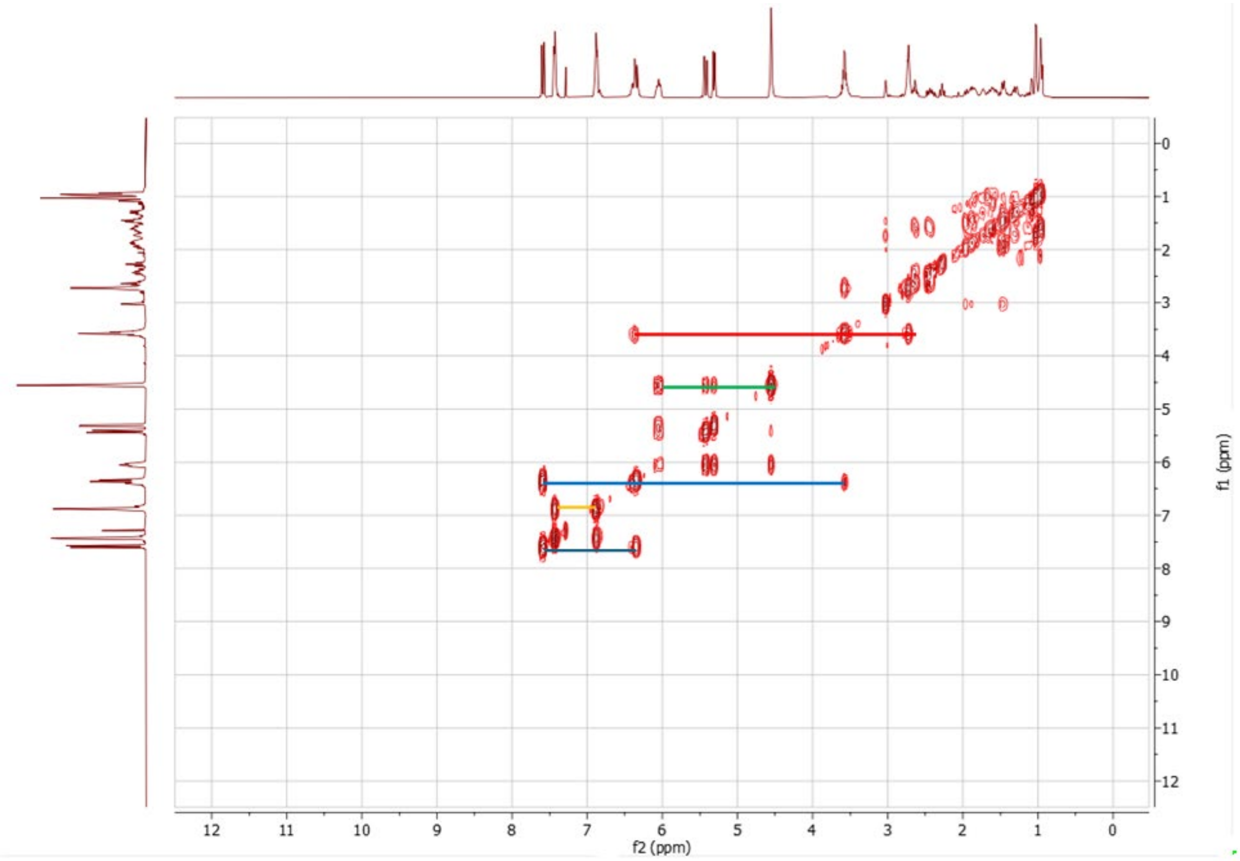

Figure S1. COSY spectrum of LCA run $CDCl_3$

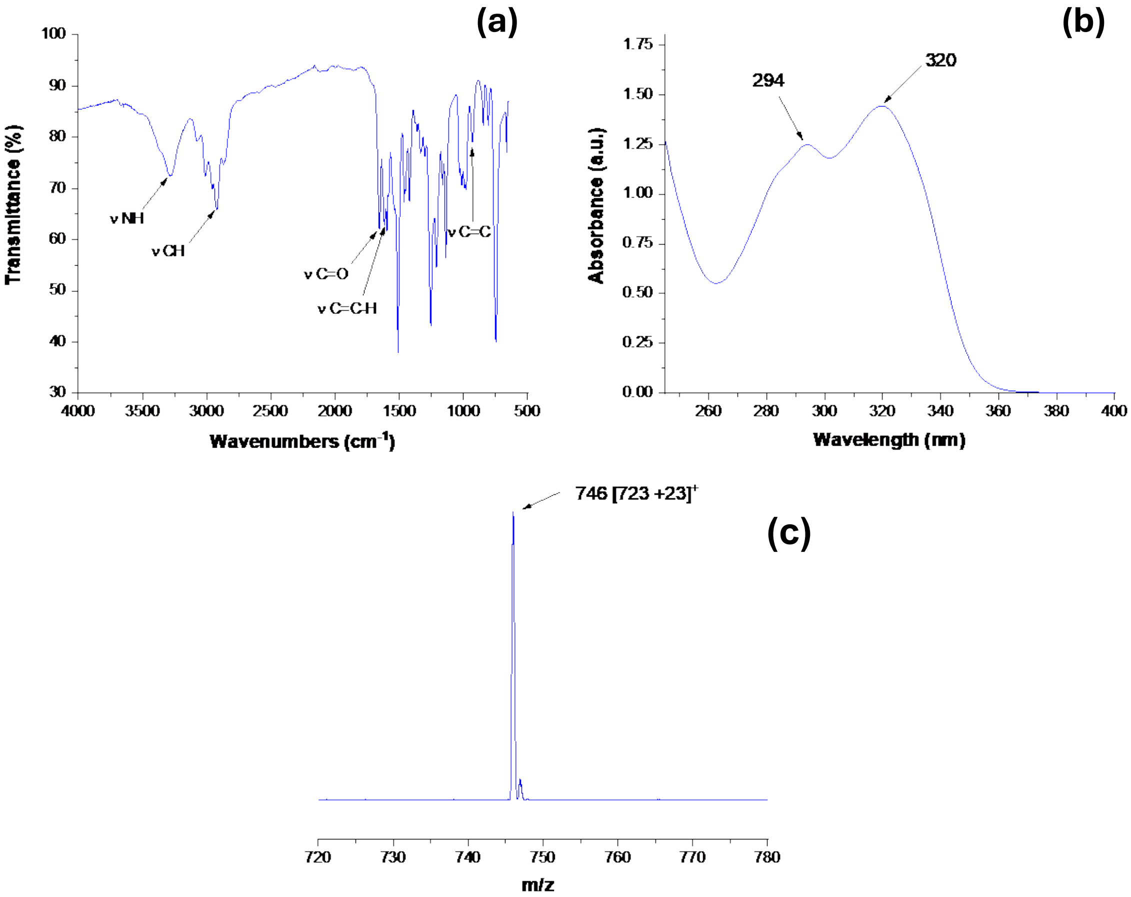

Figure S2. Chemical characterization of LFA: (a) FTIR spectrum in KBr, (b) UV-Vis spectrum in $CHCl_3$ and (c) MALDI-TOF spectrum

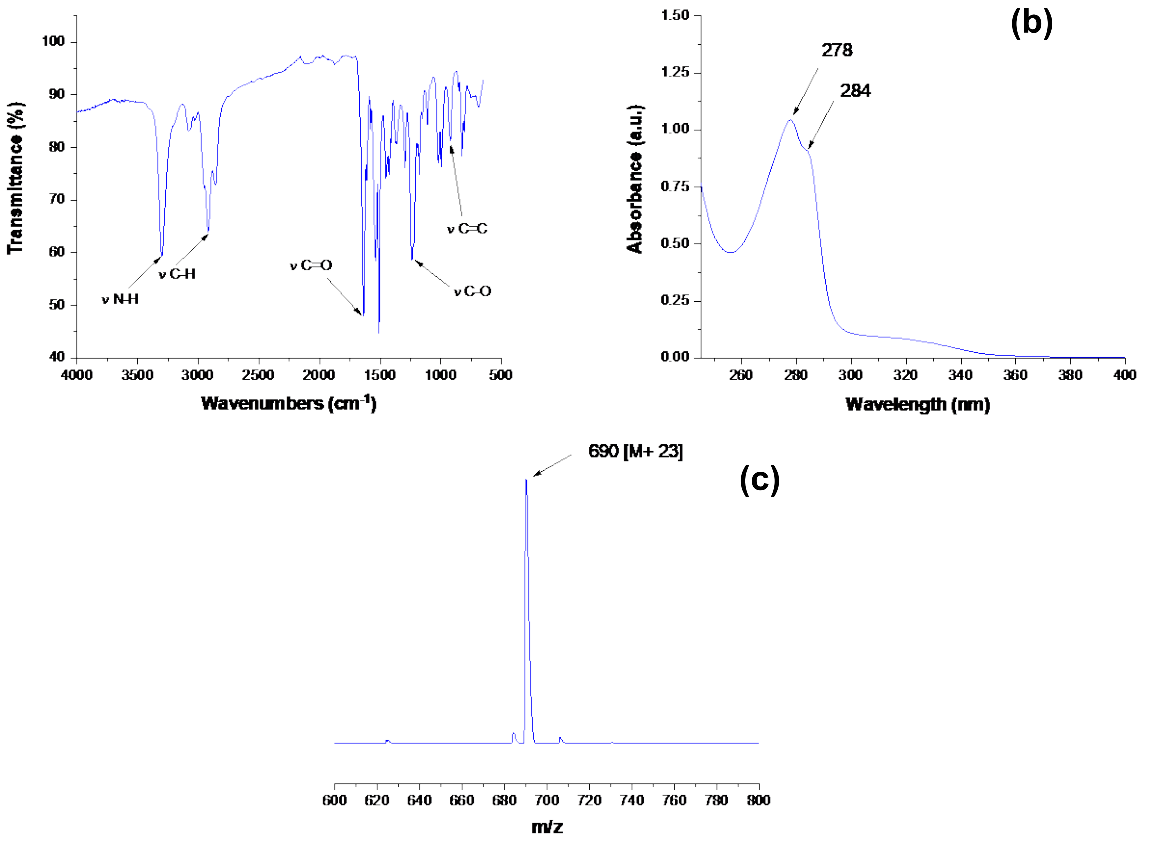

Figure S3.Chemical characterization of LPA: (a) FTIR spectrum in KBr, (b) UV-Vis spectrum in $CHCl_3$ and (c) MALDI-TOF spectrum
